# The moderating role of intrinsic motivation on the relationship between toxic leadership and emotional exhaustion

**DOI:** 10.3389/fpsyg.2022.1047834

**Published:** 2022-12-12

**Authors:** Oktay Koç, Serdar Bozkurt, Deniz Devrim Taşdemir, Ayşe Günsel

**Affiliations:** ^1^Political Science and Public Administration Department, Sinop University, Sinop, Turkey; ^2^Business Administration Department, Yildiz Technical University, İstanbul, Turkey; ^3^The Ministry of National Defense, Ankara, Turkey; ^4^Business Administration Department, Kocaeli University, Kocaeli, Turkey

**Keywords:** toxic leadership, emotional exhaustion, administrative nursing research, PLS-SEM, intrinsic motivation

## Abstract

Dysfunctional and destructive leadership behaviors have begun to be seen frequently in today’s business world. Likewise, toxic leadership, with incompetent supervision elements results with negative outputs for organizations and heavily for the employees. Employees may experience long-term stress in the work environment and develop emotional exhaustion, resulting in mental breakdown. Hence, this study aims to reveal the effects of toxic leadership on emotional exhaustion within the healthcare industry as a first step. Moreover, we also attempt to reveal the contingency of intrinsic motivation to lessen the reflections of toxic leadership on emotional exhaustion as a second step. Using PLS-SEM, we find that toxic leadership is positively associated with emotional exhaustion. Furthermore, our findings provide empirical evidence supporting the moderator role of intrinsic motivation on the relationship between toxic leadership and emotional exhaustion.

## Introduction

While many organizational behavior studies focus on traits and behaviors that make leaders effective, many leaders in professional life perform dysfunctional and destructive behaviors (Schmidt, 2008). Even though this “dark side” of leadership has been largely ignored (Reyhanoğlu and Akın, 2016), increasing expressions of displeasure by employees with the quality of the leadership they receive are addressed by such bodies as The Work Foundation and the Chartered Institute of Personnel and Development, all of which claim that workplace dysfunction and leadership toxicity may be the normal of today’s organizational life (Walton, 2007).

“Toxic leadership is a silent killer.” Like a deadly poisonous snake, toxic leaders feed on energy from competent workers, and they depress competent workers who are also creative and energetic. In so doing, they create a demoralizing, dehumanizing, and fearful business environment that paralysis the organization (Indradevi, 2016). For affected followers, toxic leadership behaviors are associated with psychological distress (i.e., anxiety and depression), emotional harm (i.e., emotional exhaustion, fear, and social isolation), and physical health problems (i.e., chronic fatigue and insomnia; Webster et al., 2016). As a very common leadership approach, particularly in “win or die” work cultures, toxic leadership results with loss of productivity, turnover, and legal problems. Toxic leadership is estimated to cost US employers $23.8 billion annually. According to a workforce consulting firm Life Meets Work report, 56% of employees complain about toxic leaders and the problematic business environment created by those leaders (Matos, 2017). Thus, toxic leadership is a negative leadership type with incompetent supervision elements (Dobbs, 2014) that creates substantial negative outputs for both organizations and employees, such as reduced employee satisfaction and commitment and increased psychological distress and burnout (Tepper, 2000; Wilson-Starks, 2003; Lipman-Blumen, 2005; Rubino et al., 2009; Aubrey, 2012; Özer et al., 2017).

Over the last 50 years, many studies are conducted on employee burnout (Maslach et al., 2001; Halbesleben and Demerouti, 2005). Burnout is “a prolonged response to chronic emotional and interpersonal stressors on the job and is defined by the three dimensions of emotional exhaustion, cynicism, and inefficacy” (Maslach et al., 2001). Even though toxicity in general meaning seems to be an important antecedent of emotional exhaustion by creating great stress on employees (Rubino et al., 2009), empirical research on emotional exhaustion from toxicity perspective are scant. More specifically, toxic leadership, as considered the dark side of leadership, also leads to emotional exhaustion in employees. Yet very little empirical research shows that emotional exhaustion is studied from the perspective of leadership on the dark side. Accordingly, this paper aims to reveal the effects of toxic leadership on emotional exhaustion. For this purpose, we consider toxic leadership as a five-dimensional construct (i.e., Abusive Supervision, Authoritarian Leadership, Narcissism, Self-Promotion, and Unpredictability) following with Schmidt (2008) study. Moreover, we also attempt to clarify the role of intrinsic motivation in the interrelationships among toxic leadership and emotional exhaustion. Intrinsic motivation is related to the intrinsic propensity to venture into the world for one’s enjoyment; to chase novelty, challenges, and learning opportunities (Gagné and Deci, 2005; Chang et al., 2015). Emotional exhaustion is a state of burnout resulting from chronic stress caused by a mismatch between a person’s needs and the workplace atmosphere (Maslach and Leiter, 2008). Such a mismatch may also be caused at least partly because of motive incongruence. Employees generally choose their job with the objectives and tasks in their job based on their goals and their self-concept. A person’s goals and self-concept refer to the motive system (Kehr, 2004). So employees with higher intrinsic motivation may be more resilient to deal with toxic leaders and they may protect themselves against emotional exhaustion by their inner motives.

**Figure 1:**
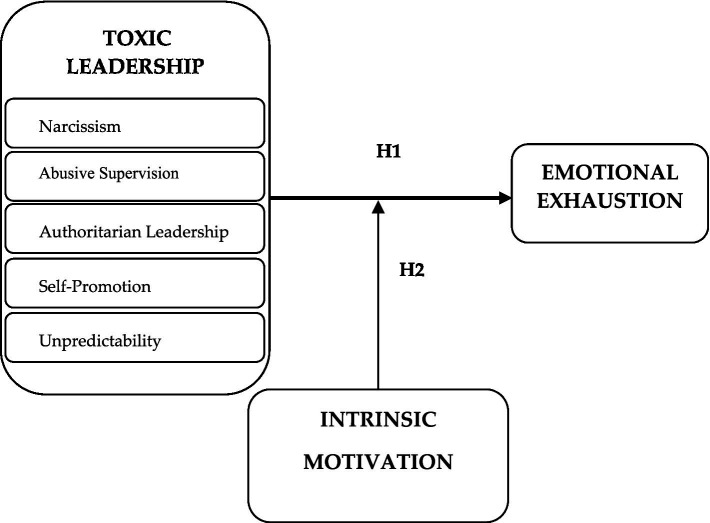
Research model.

This study is substantially guided by the following important two research questions: (i) how toxic leadership affects emotional exhaustion and (ii) do the effects of toxic leadership on emotional burnout differ by virtue of the intrinsic motivation of employees? Self-determination Theory, a macro-theory that details the origins and outcomes of human behavior, focuses mainly on motivation and personality (Deci and Ryan, 2012; Adams et al., 2017). Self-determination theory assumes that intrinsic motivation is a deeply evolved propensity to apply and extend the skills and capacities of human beings (Ryan et al., 2009), such as the individual’s capacity to cope with toxic leaders. Accordingly, this study aims to enrich the self-determination theory by addressing the shielding role of intrinsic motivation against the negative effects of toxic leadership on employees in terms of emotional exhaustion. Interestingly, the interrelationships among toxic leadership, emotional exhaustion, and intrinsic motivation as a holistic model are relatively unexplored in the literature in developing countries—just like Turkey—in particular. As far as we know, no methodological framework for such a holistic approach has been developed yet. To test our hypothesis, we selected the nurses working in private hospitals in İstanbul. The reason underneath the choice of nurses is that health institutions are convenient environments for toxic relationships as well as military organizations or political ones (Kusy and Holloway, 2009; Rosenstein, 2009; Roter, 2011). From a practical standpoint, the proposed model (Figure 1) enhances the understanding of health managers with regard to increasing emotional exhaustion and burnout of health sector employees.

## Theoretical background

### Toxic leadership

Leadership is a broadly studied concept in management and organizational behavior (OB) literature. Most of the literature abounds with studies on leadership styles that create an environment of trust that contributes to the development of their followers and the achievement of organizational goals. However, professional life encounters many examples of managers or leaders who act precisely the opposite (Bennis and Thomas, 2011). At this point, the concept of toxic leadership is coming out often in the leadership literature recently. The term toxic leadership has been used to define the leaders or managers who display maladaptive, malicious, and disgruntled behaviors, creating negative reflections on their subordinates. They also prevent information and cooperation while distrusting and demoralizing them (Kellerman, 2008; Schmidt, 2008). The word toxic, which is of Greek origin, is defined as the potential to cause disability or even death by poisoning (Agid, 1998). The term toxicity is used in many areas. For instance, “emotional toxicity” is employed to define the individuals/employees who consume the energy of others, causes negative consequences such as anxiety and distress, and leads to a problematic organizational atmosphere that is generally ineffective, inadequate, and destructive to the others (Bolton, 2005; Bacal, 2012).

Whicker (1996) introduced the concept of toxic leadership as a leadership style that is negative, humiliating, dysfunctional, maladaptive, and malicious. Recent studies consider managers who—consciously or unconsciously—damage both the organizations and the employees as toxic leaders (Lipman-Blumen, 2005; Williams, 2005). According to Wilson-Starks (2003), leaders who destroy employees’ self-esteem *via* formal authority and cause them to quit are also considered toxic leaders. Toxic leaders perform good behaviors to influence their superiors, allure people with their success, and become indispensable in the organization. This effect is not easily detectable, so toxic leaders can manipulate others to cover their destructive intentions (Lipman-Blumen, 2005). Moreover, with toxic leaders’ slow and deep moves, the organization cannot easily understand their real damage to the organization (Gangel, 2007; Reed, 2007).

Toxic behaviors may be conscious, or they may unconsciously result from incompetence. Both cases are considered within the context of toxic leadership. Because—consciously or unconsciously—the employees and the organization are exposed to demoralizing, malicious behaviors and experience the same hostile undermining climate. In both cases, selfish, destructive, bullying, and toxic behaviors toward employees are significant. Toxic leadership, which refers to the dark side of leadership, also reduces motivation and efficiency (Williams, 2005). According to Lubit (2004), toxic leaders have four key traits. The first one is narcissism with oppressive tendencies, over-controlling, and anti-social personality. The second trait is aggressiveness; toxic leaders often engage in bullying, are usually out of control, and behave angrily. The third trait is coercive, anxious, and apathetic authoritarianism. They are also arrogant, self-confident, and even control freaks, not caring about others. Bullying, disregarding the opinions of others, and the desire to expand the area of control is the fourth trait of this leadership.

Furthermore, another comprehensive study by Schmidt (2008), conducted in American Navy, puts forward a five-dimensional toxic leadership construct composed of narcissism, unpredictability, self-promotion, and authoritarian and abusive leadership. To briefly explain the characteristics of toxic leaders:

- Narcissistic leaders see themselves as superior to others. They use their legal authority to expand their position and consider that no one else has leadership, administration, or supervision skills like them. In addition, narcissistic leaders tend to expect others to admire them with the belief that they are unique (Rosenthal and Pittinsky, 2006; Reyhanoglu and Akin, 2020).
- The behaviors and moods of toxic leaders are unpredictable. They are not competent at managing their emotions—those unexpected emotional states can cause negative consequences for the organization. Also, the daily mental mood of such leaders is a source of uncertainty that may affect the organization’s emotional status. Toxic leaders may sometimes be friendly and welcoming, while they may be cruel and malicious in other times. This unpredictable mood keeps everyone on edge. They create powerless employees by making them puzzled, and no one can be sure what is coming next (Schmidt, 2008).
- Self-promoted leaders take all the credit for their team’s triumph and refer to all good results as achievements for increasing their self-interests. They reshape the work environment to improve their situation and support employees who serve their interests. Self-promoted leaders are good at impression management; they do not hesitate to imitate others while displaying their indispensability (Schmidt, 2008).
- Authoritarian leaders establish a micromanagement system in which a sole person—themselves—gathers power. Using their authority as an element of pressure on others, authoritarian leaders strictly control their employees’ every activity. They issue orders and devalue the opinions of subordinates, but when it comes to an unfavorable situation, they blame others. Authoritarian leaders do not empower their subordinates or do not allow employees to use their creative potential. Leaders of this type gather all resources for their interests and are unwilling to delegate any power. The controlling role is dominant, and they expect obedience (Cheng et al., 2004).
- Abusive leaders do not hesitate to use their employees outside of organizational tasks. They can exceed their legal edges for their own interests. Those leaders set arbitrary standards beyond administrative requirements. They can explicitly humiliate and abuse their employees, often reminding them of past failures (Tepper, 2000; Tepper, 2007). Abusive leaders make their subordinates feel insecure (Dobbs, 2014).

Based on the abovementioned characteristics, we conclude that toxic leaders try to dominate their followers instead of inspiring them. For their success, they do not consider the negative consequences of their behaviors on their subordinates and the organization. They may consume others’ energy and decrease their performance, So the work becomes more complicated, the efficiency decreases, and the positive relationships in the organization deteriorate. The employees’ well-being is damaged (Lubit, 2004) which may result in emotional exhaustion.

### Emotional exhaustion

The concept of emotional exhaustion was discussed as an explanatory element of the concept of burnout in Maslach (1982) research. Emotional exhaustion is considered to be burnout’s core dimension (Halbesleben and Bowler, 2007). Furthermore, emotional exhaustion is the central stage because it seizes the core of burnout (Shirom, 1989). Emotional exhaustion refers to the draining and depleting inner and outer energy (Anbar et al., 2007). According to Demerouti et al. (2001), emotional exhaustion results from prolonged exposure to intense physical and cognitive job demands. The overuse of feelings is one of the leading causes of emotional exhaustion (Maslach et al., 2001). Emotional exhaustion is the initiation and significant component of burnout and mainly refers to reducing emotional and physical resources (Wright and Bonett, 1997; Maslach et al., 2001). In other words, emotional exhaustion is a state of exhaustion caused by excessive psychological needs in elevated human relationships (Jackson et al., 1987). Emotional exhaustion emerges as a reaction to overwhelming emotional requests in which individuals are in intense emotional labor (Liu et al., 2020).

Emotional exhaustion is very common in professions where intense and face-to-face relations among people are inevitable (Örücü and Hasırcı, 2020). Extreme concerns and a deficit of energy cause the feeling of depleting emotional resources. As a result, commitment to achieve decreases, and job stress often increases. In such a set, it is almost impossible to manage the business requirements. Moreover, an individual with a negative mindset has great anxiety about the business environment (Cordes et al., 1997). On account of additional anxiety, emotional exhaustion not only causes an upsurge in negative emotions such as fatigue, energy loss, weakness, depressive sensation, hopelessness, anger, impatience, and restlessness, but also a decline in positive emotions such as respect, friendship, and kindness (Ersoy et al., 2001). Earlier studies also indicate that several work features such as high job requests and workloads, term anxieties, and extended working hours are likely to donate to exhaustion (Bakker et al., 2000; Maslach et al., 2001; Bekker et al., 2005). The broadly environmental consequences of emotional exhaustion are absenteeism, a decline in organizational performance due to the decrease in employee performance, and lowering productivity by expanding the employee costs of organizations (Maslach and Zimbardo, 1992; Ardıç and Polatçı, 2008).

### Intrinsic motivation

Motivation takes its roots from the Latin word “movere,” which means “to move.” Woodworth (1918) first introduced motivation as “the energy that activates the individual.” As a critical antecedent of employee efficiency and productivity, motivation is among the main issues of management and organizational behavior literature. The source of motivation is goals, and it is of great importance to encourage employees by meeting a set of expectations. The term is based on the assumption that motivation occurs when three specific conditions are met: effort, performance, and outcome.

Satisfaction with essential needs boosts motivation and drives employees to succeed much more than being forced (Ryan and Deci, 2000; Byrd et al., 2007). Motivation is a mechanism that enables employees successfully fulfill their job requirements, direct their energies to specific goals, and concentrate their efforts (Barrick et al., 2015). It emerges as a driving force and directs the individual to achieve their real aim. Zhang and Bartol (2010) argue that employees can concentrate their energies in the requested direction by reducing the influence of external issues. In this way, employees can enjoy the work while pursuing their interests and skills. Based on all those definitions above, we conclude that motivation is a meaningful way to activate the effort and desire to reach a goal and work more effectively.

In the extant literature, as a critical element for success, motivation is often considered an intrinsic drive for behaving or acting in a specific manner (Jovanovic and Matejevic, 2014). Intrinsic motivation is the desire to do something and succeed without a compelling factor. This driving force encourages employees to do something without external motives or pressure. It directs the internal reactions until the desire is achieved. Intrinsic motivation emerges when an individual does his/her task without visible rewards (Wiersma, 1992). If the context is expanded a little more, intrinsic motivation can be expressed as the individual’s decision to struggle spontaneously without external rewards or pressures (Oudeyer and Kaplan, 2007). In intrinsic motivation, the reward is the inner activity itself; it helps individuals unveil their inner power. The individual’s expectations compel them to follow their wills. The individual’s belief and the ability to work not only increase intrinsic motivation based on psychological needs but also motivate them to succeed (Cook and Artino, 2016). According to Thomas and Velthouse (1990), actions that empowerment and participation in decision-making are essential components of internal motivation. Also, letting them show creativity feels people necessary and boosts intrinsic motivation.

Furthermore, according to Mottaz (1985), creating an opportunity to use skills and provide feedback on consequences outcomes increases intrinsic motivation. As a result, intrinsic motivation is the effort to succeed without the necessity of any external influences. In professional life, tasks accomplished with intrinsic motivation will help individuals adopt and establish a positive connection with jobs and tasks (Barrick et al., 2015). Higher intrinsic motivation contributes positively to organizational achievement if the goals satisfy employees’ basic needs.

## Hypothesis development

### Toxic leadership and emotional exhaustion

Leadership studies label authoritarian, rigid, unethical, exuberant, abusive, bullying, narcissistic, paranoid, and deterrent leadership behavior and styles as toxic leadership (Pelletier et al., 2019; Schilling et al., 2022). The consequences of toxic leadership attract the attention of various management experts today. The most important reason is that, in many cases, toxic leadership has harmful effects on organizational culture, policies, programs, and organizations, as well as the employees (Vreja et al., 2016). Toxic leaders may also cause “severe problems in business life,” “a decrease in the employees’ performance in the workplace,” and “an increase in the level of emotional exhaustion and psychological problems” (Krumov et al., 2016). According to Glasø and Vie (2009), subordinates repeatedly exposed to toxic leaders suffer from frustration and emotional exhaustion. Prolonged stress also emerges as a key antecedent of emotional exhaustion (Lourel et al., 2008; Hobfoll et al., 2018). Toxic leadership, infusing toxic thoughts and creating stress, affects employee well-being and leads them to exhaustion. Many recent studies (e.g., Khakpour, 2019; Khan et al., 2019; Malik et al., 2019) reveal a positive relationship between toxic leadership and emotional exhaustion. Furthermore, Ekvall (2002) provided empirical evidence in support of the antecedent role of the climate that harbors toxic leaders for emotional exhaustion. Based on those arguments, we expect leaders or managers performing toxic leadership behaviors to be one of the main causes of emotional exhaustion among the employees. Accordingly,

*Hypothesis 1 (H1):* Toxic leadership has a positive effect on emotional exhaustion.

### Intrinsic motivation as a moderator

Intrinsic motivation is a vital descriptor of an individual’s propensity to innovate and engage in challenging tasks, develop and use their capacity, and explore and learn. Demonstrating intrinsic motivation is influenced by social and environmental factors that facilitate or weaken it (Barrick et al., 2015). Self-determination theory describes how the individual interacts with the social environment and the factors that make up the individual’s motivation. At the same time, the theory examines the influence of social and cultural backgrounds on individuals’ basic psychological needs, performance, and well-being, based on the idea that the individual is in constant dynamic interaction with the social world (Legault et al., 2017).

Intrinsically motivated individuals are at the highest level of self-determination. Individuals with intrinsic motivation pursue their behavior with pleasure and have feelings of self-satisfaction and competence. Autonomy and feelings of internal control are evident (Legault et al., 2007; Deci et al., 2017). In this context, we used self-determination theory to explain the moderator role of intrinsic motivation in the relationship between leadership and emotional exhaustion.

Managers and academics need to motivate their employees to put in more effort. We know very little about the psychological mechanisms underlying the motivational effects of toxic leadership. What is certain is that toxic leadership reduces employee motivation and productivity (Reed and Bullis, 2009). According to Meng et al. (2017), toxic leaders negatively affect intrinsic motivation through leader-member relationships. They undermine subordinates’ motivation, well-being, or job satisfaction without clearly harming the organization’s goals (Einarsen et al., 2007).

On the other hand, Koropets et al. (2020) argue that toxic leadership increases work-related stress and emotional exhaustion. Intrinsic motivation is required to reduce the negative reflections that individuals are exposed to. Moreover, Michel and Hargis (2017) find that intrinsic motivation can protect employees and shield them against toxic leadership by increasing performance. Karatepe and Tekinkus (2006) research on front-line employees shows the decreasing effect of intrinsic motivation on emotional exhaustion. Intrinsically motivated employees, motivated by their interest and satisfaction, are likely to show more interest in their work and experience less emotional exhaustion (Margaretha, 2019), while employees with low intrinsic motivation are more vulnerable to emotional exhaustion (Babakus et al., 2008).

Self-determination theory also buffers the adverse effects of a hostile work atmosphere, such as mental and physical health problems and emotional exhaustion (Iso-Ahola and Park, 1996). Intrinsic motivation is widely accepted as an internal power to cope with emotional exhaustion (Kim, 2015). Thus, while toxic leadership creates a negative work atmosphere which ultimately leads to adverse consequences, intrinsic motivation may put barriers to the negative effects of toxic leadership. We expect employees with high intrinsic motivation to be more resilient to the negative reflections of toxic leadership, such as emotional exhaustion. Accordingly;

*Hypothesis 2 (H2):* The higher the intrinsic motivation the weaker the relationship between toxic leadership and emotional exhaustion.

## Materials and methods

This section comprises our methodological approach taken for data collection, sampling, and analysis. Our methodological approach based on qualitative techniques in both data collection and analysis on the one hand and in sampling convenient sampling on the other hand is explained below.

### Research design

In the following sections, we describe our approach in research design from a broader perspective and more specifically in measures, sampling, and analysis. Our sample set is selected from nurses working at different hospitals by using convenient sampling method. In the analysis, we used PLS-SEM technique.

#### Measures

To test the above hypotheses, multi-item scales adopted from prior studies for the measurement of constructs were used. Each construct was measured using a 5-point Likert scale ranging from “strongly disagree” (1) to “strongly agree” (5). Toxic leadership as a composite second-order variable composed of five sub-dimensions—abusive supervision, authoritarian leadership, narcissism, self-promotion, and unpredictability—was measured with a scale consisting of 30 questions adapted from Schmidt (2008). “Ridicules subordinates” (abusive supervision), “Will ignore ideas that are contrary to his/her own” (authoritarian leadership), “Has a sense of personal entitlement” (narcissism), “Denies responsibility for mistakes made in his/her unit” (self-promotion), and “Expresses anger at subordinates for unknown reasons” (unpredictability) are examples of the toxic leadership scale items.

To measure emotional exhaustion, 9-item emotional exhaustion scale is adapted from Maslach Burnout Inventory, originally developed by Maslach and her colleagues (Maslach et al., 2001). “I feel emotionally exhausted because of my work” and “I feel worn out at the end of a working day” are exemplar items.

Finally, to measure intrinsic motivation, 6 items are adopted from Intrinsic Motivation Inventory,” the perceived competence dimension of intrinsic motivation scale, originally developed by Ryan (1982). “I think I am pretty good at this work” and “I think I did pretty well at this work, compared to other employees” are exemplar items.

#### Sampling

The aim of this paper is to describe and analyze the mutual relationships between toxic leadership, emotional exhaustion, and intrinsic motivation among the nurses. In order to empirically investigate the hypotheses, 600 nurses employed in healthcare institutions located in Istanbul are chosen as the target sample based on their accessibility. First, the selected 600 nurses were contacted by telephone and the aim of the study was explained to them. Of the 600 nurses contacted, 198 agreed to participate in our study. Out of the nurses that members to participate, 142 employees completed the survey. After careful examination, all the incomplete returns with the missing data were discarded, leaving 133 responses for analysis. PLS-SEM, on the other hand, can be used in studies with small samples, in cases where normality cannot be achieved, and in samples with a reasonable number of missing data (Hair et al., 2016). Depending on this, we used PLS-SEM with our relatively small sample.

The findings are based on data from a convenience sample of which 92% of the participant nurses (*n* = 122) were female and 97 of the participants were married (73%). 82% of participants had bachelor or higher degrees; 67% of the participants (*n* = 89) were working in public owned healthcare institution. Forty-two of the participants (32%) were 28–35 years old. Moreover, 72% of the participants (*n* = 96) were employed in general hospitals with more than 200 beds and 62% of the sample (*n* = 82) had a total tenure of 5–10 years.

#### Analysis

We used PLS-SEM technique to test our model based on several considerations. First, according to Fornell and Cha (1994), PLS avoids many of the restrictive assumptions underlying maximum likelihood techniques and ensures against improper solutions and factor indeterminacy. PLS-SEM does not make any distributional assumptions regarding the inDISators or error terms (Hair et al., 2016). Indeed, PLS is a latent variable modeling technique that incorporates multiple dependent constructs and explicitly recognizes measurement error. Second, PLS is insensitive to sample size considerations and proper for any sample sizes over thirty in contrast with covariance bases SEM techniques (Fornell and Cha, 1994; Hair et al., 2013). Since our sample is composed of 133 respondents (*n* = 133 nurses) PLS-SEM seems to be a proper technique for this paper (Hair et al., 2016). Moreover, PLS handles both reflective and formative constructs (Hair et al., 2013).

## Results

### Measurement validation

In this study, following Kleijnen et al. (2007), we used reflective indicators for all our constructs. To assess the psychometric properties of the measurement instruments, we estimated a null model with no structural relationships. We evaluated reliability using composite scale reliability (CR), Cronbach’s alpha, and average variance extracted (AVE). For all measures, PLS-based CR is well above the threshold value of 0.70, Cronbach’s alpha goes beyond the threshold value of 0.70, and AVE exceeds the 0.50 threshold value for all first-order constructs. In addition, we evaluated convergent validity by inspecting the standardized loadings of the measures on their respective constructs and found that all measures exhibit standardized loadings that exceed 0.60.

Table 1 shows the correlation among all seven variables that provide further evidence of discriminant validity. To fully satisfy the requirements for discriminant validity, AVE for each construct should be expected to be greater than the squared correlation between constructs (Fornell and Cha, 1994). Such results suggest that the items share more common variance with their respective constructs than any variance the construct shares with other constructs (Howell and Avolio, 1993). In the model, none of the inter-correlations of the constructs exceeded the square root of the AVE of the constructs (see Table 1). In addition, we evaluated convergent validity by inspecting the standardized loadings of the measures on their respective constructs and found that all measures exhibit standardized loadings that exceed 0.60.

**Table 1:** Reliability and validity.

		α	AVE	CR	1	2	3	4	5	6	7
1	Emotional exhaustion	0.95	0.78	0.96	0.88						
2	Intrinsic motivation	0.80	0.50	0.86	−0.45	0.71					
3	Narcissism	0.92	0.76	0.94	0.30	−0.30	0.87				
4	Abusive supervision	0.94	0.77	0.95	0.43	−0.37	0.66	0.88			
5	Authoritarian leadership	0.89	0.69	0.92	0.36	−0.39	0.68	0.74	0.83		
6	Self-promotion	0.96	0.80	0.97	0.39	−0.40	0.76	0.76	0.76	0.90	
7	Unpredictability	0.93	0.72	0.94	0.41	−0.29	0.6	0.64	0.66	0.67	0.85

Diagonals show the square root of AVEs. CR, composite reliability; AVE, average variance extracted; α, Cronbach’s alpha.

**Figure 2:**
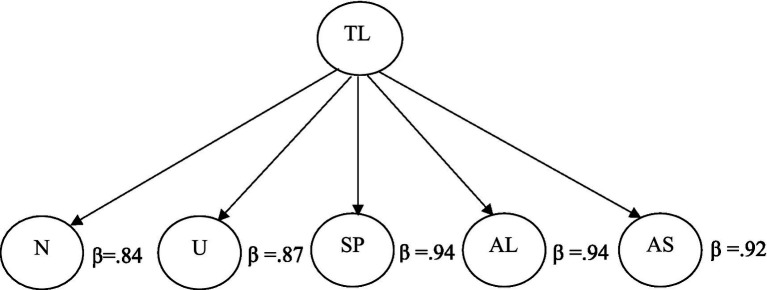
Second-order factor analysis of toxic leadership. TL, toxic leadership; N, narcissism; U, unpredictability; SP, self-promotion; AL, authoritarian leadership; AS, abusive supervision.

Moreover, since we used toxic leadership as a composite variable, composed of 5 dimensions—narcissism, unpredictability, self-promotion, authoritarian, and abusive leadership—we also performed a second-order factor analysis. Figure 2 shows the standardized regression loadings of those given five constructs. As seen in Figure 2, all five constructs exceed a standardized loading of over 0.60. This result suggests that toxic leadership as a five-construct second-level variable is significantly predicted by narcissism, unpredictability, self-promotion, authoritarian, and abusive leadership.

### Hypothesis testing

The PLS (Partial Least Squares) approach (Chin et al., 2003) and the bootstrapping re-sampling method were used by computing the SmartPLS 3.0 software program to estimate the main effects as well as the interaction ones, and to test the hypothesis and predictive power of our proposed model (see Figure 1). T-statistics were estimated for all coefficients, based on their stability across the sub-samples, so as to define the links that were statistically significant. The path coefficients and their associated t-values showed the direction and impact of each hypothesized relationship. Following the recommendation of Chin et al. (2003), a hierarchical approach for testing the hypotheses was used: a model with the main effects (and covariates) was assessed, after which the interaction effects were added.

First, regarding the direct effects, our findings show that toxic leadership is positively and significantly associated with emotional exhaustion (β = 0.28 *p* < 0.01), supporting *H*1.

Second, A two-step procedure was used to address the hypothesis concerning the moderating effects of intrinsic motivation (Chin et al., 2003). The PLS approach enables explicit calculation of the standardized latent variable scores after saving the obtained results (Chin, 2001). Here, each item of toxic leadership and intrinsic motivation was standardized. Based on this procedure, the standardized question items were multiplied. The results demonstrated a negative interaction effect of intrinsic motivation (β = −0.18, *p* < 0.05) on the relationship between toxic leadership and emotional exhaustion, supporting *H*2 (Table 2).

**Table 2:** Hypothesis testing results.

Hypothesis	Relationships	*β*	Path results
*H*1	Toxic Leadership➔ Emotional exhaustion	0.28**	Supported
*H*2	Toxic leadership*Intrinsic motivation➔ Emotional exhaustion	−0.18*	Supported

*p < 0.05, **p < 0.01.

### Structural model

In order to validate the PLS-SEM approach, various quality scores, such as the coefficient of determination (*R*^2^; Chin, 2001) and the Goodness-of-Fit Index (GoF; Fornell and Lacker, 1981) are being considered. The *R*^2^ values of the endogenous constructs are used to evaluate the model fit and indicate how well data points fit a line or curve (Chin, 2001; Tenenhaus et al., 2005). As suggested by Chin (2001), the categorization of *R*^2^ values is small (0.02 ≤ *R*^2^ < 0.13), medium (0.13 ≤ *R*^2^ < 0.26), or large (0.26 ≤ *R*^2^). In addition, GoF is employed to globally evaluate the overall fit of the model, seeking a concordance between the performance of the measurement and the structural model, as well as being consistent with the geometric mean of the average commonality and the average *R*^2^ of endogenous latent variables. GoF ranges between 0 and 1, where a higher value represents better path model estimation. In line with the effect sizes for *R*^2^, using 0.5 as a cut-off value for commonality (Fornell and Lacker, 1981), threshold values for the GoF criteria are categorized as small (0.1 ≤ GoF < 0.25), medium (0.25 ≤ GoF < 0.36), or large (0.36 ≤ GoF) effect sizes. Table 3 shows *R*^2^ and GoF values as the fit measures of the structural model. In accordance with the categorization of *R*^2^ effect sizes, the effect size for emotional exhaustion (*R*^2^ = 0.33) is large According to another fit measure, the result of GoF was 0.31 revealing a medium-sized fit (see Table 3).

**Table 3:** Structural model.

Fit measures	Endogenous constructs	
*R* ^2^	Emotional exhaustion	0.33
GoF		0.31

GoF = √ Average communality × Average *R*^2^.

## Discussion

Toxic leadership is progressively becoming prevalent in today’s organizations in general, in healthcare organizations in particular (Yayla and Eskici İlgin, 2021). Since the last decade, the extant literature has largely examined toxic leadership to understand its antecedents and consequences at the organizational level. However, little is known about the mechanisms that protects employees against the negative reflections of toxic leadership. The current research studied the contingency of intrinsic motivation on the toxic leadership-emotional exhaustion relationship within the healthcare domain. This relationship was foreseen for employees with higher intrinsic motivation are less likely to suffer from emotional exhaustion, even they are exposed to toxic leadership. The results fully supported this prediction.

Theoretical arguments on toxic leadership draw attention to the negative consequences of this leadership style on both organizational and individual levels (Schmidt, 2008; Glasø and Vie, 2009; Pelletier, 2010; Schyns and Schilling, 2013; Webster et al., 2016; Labrague et al., 2020). We empirically show that nurses exposed to toxic leadership behaviors are likelier to suffer from emotional exhaustion. This result is consistent with previous research conducted at the individual level (Ekvall, 2002; Khakpour, 2019; Khan et al., 2019). Second, we demonstrate also that nurses with higher intrinsic motivation are less prone to emotional exhaustion, even if they work with toxic leaders. Intrinsic motivation is the effort to succeed without the necessity of any external influences (Barrick et al., 2015; Legault et al., 2017). As an intrinsic drive for behaving or acting in a specific manner, intrinsic motivation directs the individual to focus on their task, ignoring the task environment. So, intrinsic motivation, in a way, serves as a protective shield against toxic leaders or managers (Michel and Hargis, 2017). To put it shortly, the employees with higher intrinsic motivation are more protected. So, they suffer less from emotional exhaustion or other negative influences of toxic leadership. This finding contributes to self-determination theory by providing empirical evidence that intrinsic motivation as a trait has the potential to extend the employee’s capacity to cope with toxic leaders, withstand accumulated stress in the work environment, and to feeling emotionally worn out and drained as a result of accumulated stress, and to protect themselves from feeling emotionally worn out and exhausted as a result of that accumulated stress.

Overall, our model shows that toxic leadership is an antecedent of emotional exhaustion in healthcare employees, while intrinsic motivation negatively conditions the impact of toxic leadership on emotional exhaustion: The higher the intrinsic motivation, the weaker the toxic leadership-emotional exhaustion relationship.

## Managerial implications

Managers exhibiting toxic leadership behaviors can cause severe problems both for organizations and employees. Such that, about one in five bosses engages in toxic behavior in the upper echelons of the corporate world, revealing the fact that toxicity problem is gradually destroying their subordinates’ morale, motivation, and self-esteem. Since emotional exhaustion also leads to work alienation, intention to quit, and decrease in job satisfaction, organizational commitment, and job performance, organizations in general and healthcare organizations in particular should take the necessary steps to diagnose and to prevent toxic leadership behavior in the workplaces. As said “prevention is better than cure,” if diagnosed at right time the treatment becomes easier.

On the other side, intrinsic motivation emerges as a protective shield that protect employees against the negative effects of toxic leadership, such as emotional exhaustion. Hence, organizations, whether public or private, should also attempt to find and implement new ways to increase employees’ intrinsic motivation, mainly when tools for increasing extrinsic motivation are unavailable. To do that, for example, utilizing supportive human resources practices, such as training and development, reward systems, recruitment, performance appraisal, and employee participation, can highly be recommended and should seriously be considered by relevant managers.

## Limitations and future research

There are some methodological limitations to this study. First, we conducted this research in a specific service industry context, i.e., healthcare organizations. Thus, researchers should be cautious when generalizing the results to different industries. The sample was relatively small (*n* = 133); a larger sample might better represent the population of the healthcare industry. Moreover, we performed a cross-sectional design with questionnaires. Although surveying is a large and growing area of research in the natural environment, the method used (only a questionnaire) may not deliver objective and comprehensive regarding the utilization of intrinsic motivation, radiating from somewhere deep inside, against toxic leadership, which is an inherently dynamic phenomenon. However, it should be mentioned that, as a cross-sectional field study, this research provides some evidence of associations. Specifically, our research is prone to common method bias since the same respondents answered the dependent variable that answered the independent variable, in a cross-sectional manner. We checked this potential problem by following Kock (2015) suggestion who argues that variance inflation factors (VIFs) higher than 3.3 is an indication of common method bias. The results of our VIFs analysis demonstrate that the VIFs values range between 1.37 and 2.34, i.e., the values are lower than the suggested threshold of 3.3. Thus, the proposed model does not appear to be affected by common method bias. Moreover, self-reported data is another problem due to the social desirability concern. Longitudinal research may go beyond this concern. Future studies may find it fruitful to examine the contingency of extrinsic motivation in addition to the intrinsic one in the toxic leadership-emotional exhaustion relationship to find out which motivational source is better at protecting employees against the negative consequences of toxic leadership. Moreover, future studies may consider using other individual outputs (i.e., psychological resilience, vengeful behaviors, cynicism, silence, or counter-productive behaviors) to extend the research model.

Finally, we studied the contingency of intrinsic motivation in toxic leadership and emotional exhaustion relationship within the healthcare industry, specifically during the COVID-19 pandemic; future studies may find it useful to examine these relationships in other industrial contexts, such as banking and finance, military, manufacturing, and high-tech industries.

## Data availability statement

The raw data supporting the conclusions of this article will be made available by the authors, without undue reservation.

## Ethics statement

Ethical review and approval was not required for the study on human participants in accordance with the local legislation and institutional requirements. Written informed consent for participation was not required for this study in accordance with the national legislation and the institutionalrequirements.

## Author contributions

AG, SB, DT, and OK: conceptualization, investigation, and writing—review and editing. AG and OK: methodology, validation, data curation, and writing—original draft preparation. AG, SB, and DT: software and visualization. AG: formal analysis. OK: resources, supervision, and project administration. All authors have read and agreed to the published version of the manuscript.

## Conflict of interest

The authors declare that the research was conducted in the absence of any commercial or financial relationships that could be construed as a potential conflict of interest.

The reviewer AG declared a shared affiliation with the author AG to the handling editor at the time of review.

## Publisher’s note

All claims expressed in this article are solely those of the authors and do not necessarily represent those of their affiliated organizations, or those of the publisher, the editors and the reviewers. Any product that may be evaluated in this article, or claim that may be made by its manufacturer, is not guaranteed or endorsed by the publisher.
